# Strepsiptera of Canada

**DOI:** 10.3897/zookeys.819.23851

**Published:** 2019-01-24

**Authors:** Jakub Straka

**Affiliations:** 1 Department of Zoology, Charles University, Viničná 7, CZ-128 44, Praha 2, Czech Republic Charles University Prague Czech Republic

**Keywords:** biodiversity assessment, Biota of Canada, Strepsiptera, twisted-wing parasite

## Abstract

In Canada, the order Strepsiptera consists of 27 known species representing five families: Corioxenidae (1 species), Elenchidae (1 species), Halictophagidae (5 species), Stylopidae (15 species), and Xenidae (5 species). These totals represent an increase of 21 species since the 1979 assessment. Half of these species represent unpublished records recently discovered by study of stylopized hosts in museum collections and DNA barcoded species. It is estimated that as many as 19 more species will eventually be discovered in Canada. DNA barcode sequences are available for 4 Canadian species. The fauna of Canada is poorly surveyed and there is a need to fill knowledge gaps with increased examination of museum specimens for stylopized hosts, broader field surveys (including use of pheromone-baited traps), and more effort to obtain DNA samples.

The order Strepsiptera, commonly known as twisted-wing parasites, are all endoparasites of other insects, especially those in the orders Hemiptera, Hymenoptera, and Zygentoma (Kinzelbach 1978). Strepsiptera have been often considered rare (Campbell 1979) based on scarcity of collection records; however, this is only an illusion as recent advances in understanding the biology of this group, including isolation of sex pheromones for some species (Straka et al. 2011, Cvačka et al. 2012, Lagoutte et al. 2013, Hrabar et al. 2015), has revealed that they are more common than previously thought. General information about the phylogenetic history of the order was reviewed by Pohl and Beutel (2013), morphology was comprehensively described by Kinzelbach (1971), and various aspects of their biology was summarized by Riek (1970), Kinzelbach (1978), Kathirithamby (2009), and Straka et al. (2011).

The status of this taxon and its relationship to other insects has been a topic of considerable debate (Pohl and Beutel 2013). For instance, when the Canadian diversity of this group was reviewed by Campbell (1979), he classified the taxon as a superfamily (i.e., Stylopoidea) of Coleoptera, though others had previously considered the group to be a separate order (e.g., Pierce 1964). The ordinal status of this group is now well accepted, and the relationship of Strepsiptera to other insect orders has been recently clarified by phylogenomic research which showed that the order is a sister lineage to the Coleoptera (Misof et al. 2014).

Worldwide, this order contains about 600 described species in nine extant families (Pohl and Beutel 2005, Bravo et al. 2009, Kathirithamby et al. 2012). The first comprehensive review of world Strepsiptera (Pierce 1909) included all species known from North America to that date. The North American species, including descriptions of new species since Pierce (1909), were later published and reviewed by Bohart (1941), Kinzelbach (1971) and Kathirithamby and Taylor (2005). These publications focused on species known mainly from the United States of America, but also included some Canadian records and species described from Canadian material. Several species such as *Stylopsleechi* Bohart, *Loaniacanadensis* Kinzelbach, and *Stenocranophiluscanadensis* Kinzelbach were described exclusively from Canada (Bohart 1941, Kinzelbach 1970, 1971).

Campbell (1979) reported only six species of Strepsiptera (from the family Stylopidae) from Canada, but predicted that an additional 10 species would likely be found in the country. Peck (1991) published the first checklist of Strepsiptera from Canada that included 11 species in three families (Peck 1991); however, he overlooked the record of *Xenospeckii* Kirby from British Columbia (Leech 1966). Subsequently, Kenner (2002) reported *Stylopsshannoni* (Pierce) from British Columbia, resulting in a total of 13 species of Strepsiptera reported from Canada.

As a result of examination of material from the Canadian National Collection of Insects, Arachnids and Nematodes (Ottawa, Ontario, Canada) and the Kansas University Natural History Museum (Lawrence, Kansas, USA), an additional 13 new Canadian species records have recently been discovered (J Straka unpubl. data), 10 from Stylopidae and three from Xenidae. One more species new to Canada was obtained from data collected in Barcode of Life Data System (BOLD; Ratnasingham and Hebert 2007). This species from BOLD belongs to the family Elenchidae, which has never been reported from Canada before. Thus, in total, 27 species from five families of Strepsiptera are now known from Canada, 15 of which are in the Stylopidae (Table 1). This represents more than four times the number of species reported by Campbell (1979), and greatly exceeds his prediction of the number of species likely to be in Canada. There are no known non-native species in the Canadian fauna. It is estimated that 19 more species of Strepsiptera will eventually be found in Canada, either by discovery of overlooked species, expansion of species in the northern United States of America into adjacent parts of southern Canada, or recognition of sibling species within currently known species. The detection of cryptic sibling species will be enhanced by DNA barcoding and application of phylogenetic reconstructions based on DNA (Jůzová et al. 2015, Straka et al. 2015a, b). Three Barcode Index Numbers (BINs) are assigned for Canadian species (Ratnasingham and Hebert 2013). One more Canadian species has already been barcoded but no BIN has been assigned to this species (Jůzová et al. 2015, Straka et al. 2015b).

**Table 1.** Census of Strepsiptera in Canada.

Taxon^1^	No. species reported in Campbell (1979)	No. species currently known from Canada	No. BINs^2^ available for Canadian species^3^	Est. no. undescribed or unrecorded species in Canada	General distribution by ecozone^4^	Information sources^5^
**Suborder Stylopidia**						
Corioxenidae	0	1	0	1	Boreal Shield	Kinzelbach 1970, Peck 1991
Elenchidae	0	1	1	4	Boreal Shield, Montane Cordillera	BOLD
Halictophagidae	0	5	1	4	Prairies, Boreal Shield, Boreal Plains	Bohart 1941, Kinzelbach 1971, Peck 1991; specimens in CNCI
Stylopidae	6	15	0 (1)	6	most ecozones except Arctic	Pierce 1909, 1919, Bohart 1941, Peck 1991, Kenner 2002; specimens in CNCI and KUNHM
Xenidae	0	5	1	4	Atlantic Maritime, Mixedwood Plains, Boreal Shield, Prairies, Pacific Maritime, Western Interior Basin, Montane Cordillera	Peck 1991, Kenner 2002; specimens in CNCI and KUNHM
**Total**	**6**	**27**	**3 (1)**	**19**		

^1^Classification follows that indicated in Pohl and Beutel (2005). ^2^Barcode Index Number, as defined in Ratnasingham and Hebert (2013). ^3^The number in parentheses represents number of barcoded species for which BINs have not yet been assigned. ^4^See figure 1 in Langor (2019) for a map of ecozones. ^5^BOLD: Barcode of Life Data System (http://www.boldsystems.org; Ratnasingham and Hebert (2007)); CNCI: Canadian National Collection of Insects, Arachnids, and Nematodes; KUNHM: Kansas University Natural History Museum.

In general, the strepsipteran fauna of Canada has not been well surveyed and all regions will benefit from increased sampling. As a starting point, it is recommended that pinned collections of common host groups, e.g., bees, wasps, leafhoppers and true bugs, be examined to locate stylopized individuals. As well, increased effort to obtain DNA barcodes as well as genome sequencing of Canadian Strepsiptera is needed. Finally, a sex pheromone of Strepsiptera that has been isolated and synthesized (Cvačka et al. 2012, Lagoutte et al. 2013, Hrabar et al. 2015) may be used in lures attached to traps to attract males, thereby aiding field sampling efforts.
